# Long-term spatial distributions and trends of the latent heat fluxes over the global cropland ecosystem using multiple satellite-based models

**DOI:** 10.1371/journal.pone.0183771

**Published:** 2017-08-24

**Authors:** Fei Feng, Xianglan Li, Yunjun Yao, Meng Liu

**Affiliations:** 1 State Key Laboratory of Remote Sensing Science, College of Global Change and Earth System Science, Beijing Normal University, Beijing, China; 2 State Key Laboratory of Remote Sensing Science, School of Geography, Beijing Normal University, Beijing, China; 3 Institute of Geographic Sciences and Natural Resources Research, Chinese Academy of Sciences, Beijing, China; 4 University of Chinese Academy of Sciences, Beijing, China; University of Vigo, SPAIN

## Abstract

Estimating cropland latent heat flux (LE) from continental to global scales is vital to modeling crop production and managing water resources. Over the past several decades, numerous LE models were developed, such as the moderate resolution imaging spectroradiometer LE (MOD16) algorithm, revised remote sensing-based Penman–Monteith LE algorithm (RRS), the Priestley–Taylor LE algorithm of the Jet Propulsion Laboratory (PT-JPL) and the modified satellite-based Priestley-Taylor LE algorithm (MS-PT). However, these LE models have not been directly compared over the global cropland ecosystem using various algorithms. In this study, we evaluated the performances of these four LE models using 34 eddy covariance (EC) sites. The results showed that mean annual LE for cropland varied from 33.49 to 58.97 W/m^2^ among the four models. The interannual LE slightly increased during 1982–2009 across the global cropland ecosystem. All models had acceptable performances with the coefficient of determination (R^2^) ranging from 0.4 to 0.7 and a root mean squared error (RMSE) of approximately 35 W/m^2^. MS-PT had good overall performance across the cropland ecosystem with the highest R^2^, lowest RMSE and a relatively low bias. The reduced performances of MOD16 and RRS, with R^2^ ranging from 0.4 to 0.6 and RMSEs from 30 to 39 W/m^2^, might be attributed to empirical parameters in the structure algorithms and calibrated coefficients.

## Introduction

Latent heat flux (LE) plays a key role in the energy and water cycles in agricultural ecosystems [1, 2]. a large number of studies have shown that LE is a vital variable for developing precise irrigation scheduling and enhancing water use efficiency in agricultural production due to the close relationship between soil water depletion and the rate of evapotranspiration (equivalent to LE) [3–6]. Quantifying water flux and energy exchange is vital for identifying cropland soil water, estimating crop factors and modeling crop production[7].

Numerous models for estimating LE have been developed, ranging from relatively simple and empirical models to move complex process level mechanistic models. The models can be grouped into the following three classes.

(1) Empirical models; Empirical models for which extensive efforts were made to seek the relationships between the LE and environmental control factors. Based on long-term observations of the global LE, Wang et al., 2007 [8] used the structure pattern of the Penman equation to develop an empirical model with the inputs of surface incident solar radiation, aerodynamic air temperature and surface skin temperature. Similar empirical correlations could also be found in crop coefficient methods, which link LE to potential LE, with empirical parameters based on vegetation conditions. Gordon et al. (2005) [9] used crop coefficient methods to estimate the global monthly vapor flow of different vegetation types.

(2) Mechanistic models; Mechanistic models describes the physical process of LE from soil surface and vegetative surface to atmosphere such as the Penman-Monteith (P-M) equation [10]. The MODIS LE product algorithm [11, 12] is an example of P-M-based model. Based on the Penman equation, many empirical or mechanistic methods were developed in the past decades. Priestley et al. (2009) [13] presented a simplified version method for when surface areas are generally wet. Fisher et al. (2008) [14] used this equation with atmospheric (RH and saturation vapor pressure deficit (VPD)) and eco-physiological (FPAR and LAI) to actual ET (ET, equivalent to LE).

(3) Fusion methods, which include multiple models based on assimilation or fusion methods. Liu et al. (2013) [15] used data assimilation schemes with the Ensemble Kalman Filter algorithm to improve the sensible and latent heat flux prediction of the community land model (CLM). Pipunic et al. (2008) [16] found that the extended Kalman assimilation filter could improve LE estimation. Yao et al. (2014) [17] used the Bayesian average model to integrate five LE models using ground observation and satellite data. Although these methods were widely used to estimate regional or global LE, the results might differ substantially due to different model structures and empirical input parameters.

Previous studies mainly concentrated on evaluating cropland LE at the regional scale, with few ground observations. Yang et al. (2014) [18] calculated the latent heat flux of the semi-arid farmland in the Loess Plateau. The results showed that the diurnal variation of LE had a peak value in August (151.4 W/m^2^) and that radiation is the leading control factor of farmland LE in the Loess Plateau. Ding et al. (2015) [19] developed a semi-empirical method-based Priestley-Taylor model with leaf area, soil moisture, mulching fraction and leaf senescence to estimate the crop coefficient and evapotranspiration in an irrigated maize field with mulching in Northwest China. Allen et al. (2000) [20] used the FAO-56 “dual” crop coefficient method to calculate daily ET at a cotton field site and a farm site in Turkey. Ershadi et al. (2014) [21] compared four LE models, including the Surface Energy Balance System model (SEBS), single-source Penman-Monteith model (P-M), advection-aridity model (AA) and Priestly-Taylor model, over a cropland ecosystem based on FLUXNET sites. Byun et al. (2014) [22] compared SEBS and modified SEBS at a rice paddy cropland site. Singh and Senay (2015) [23] compared Mapping Evapotranspiration at high Resolution with Internalized Calibration (MERTIC), the Surface Energy Balance Algorithm for Land (SEBAL), SEBS, and the Operational Simplified Surface Energy Balance (SSEBop) model for three AmeriFlux cropland sites in Mead, Nebraska. Complex land surface condition such as surface topography, changes of regional environment, model structures and lack of local data calibration often limited the comparison of LE models at the global scale. However, accurate global estimation of LE is essential for quantification of the water balance for water resources management and optimizing crop production [22]. Therefore, long-term spatial distributions and trends of LE based on different LE models in cropland ecosystems at the global scale under various climatic conditions are still needed.

In the present study, we evaluated four LE models, including MODIS Algorithm (MOD16), Revised Remote Sensing-Based Penman–Monteith LE Algorithm (RRS), Priestley–Taylor Algorithm (PT-JPL) and Modified Satellite-Based Priestley–Taylor Algorithm (MS-PT), using the LE measurements from thirty-four eddy covariance sites over cropland ecosystems, and results from temporal trend and spatial distribution of the estimated cropland LE by satellite-based observations. The objectives of this study were as follows: (1) compare LE algorithm performances over cropland ecosystems based on 34 flux tower sites provided by the EC towers network and (2) analyze the differences in the temporal and spatial patterns of LE over the global cropland ecosystem during 1982~2010.

## Datasets

### 2.1. Ground EC data

Ground data collected from 34 EC towers of FLUXNET, Asiaflux, Ameriflux were used for the analysis. We used the FLUXNET data provided by FLUXNET community (http://fluxnet.fluxdata.org/data/la-thuile-dataset/). The radiation data included continuous series of half-hourly incident solar radiation (Rs, W/m^2^), surface net radiation (Rn, W/m^2^), sensible heat flux (H, W/m^2^), latent heat flux (LE, W/m^2^) and ground heat flux (G, W/m^2^). The meteorological data included continuous series of half-hourly incident relative humidity (RH), air temperature (Ta, K), diurnal air temperature range (ΔT, K), wind speed (Ws, m/s) and vapor pressure (e, Pa). The meteorological data and radiation data provided input variables, while ground-measured LE were used as the validation [24, 25]. We converted the half-hourly data into daily data. Quality control and gap-filling techniques were utilized to strengthen the quality of the data sets. The tower sites covered main cropland ecosystems over Europe, Asia and America. The data were selected to cover the period of 2000–2009, with at least one year of data available (Table 1).

**Table 1:** Characteristics of validation data at the EC sites.

Site Name	country	lat^a^	lon^a^	year
BE-Lon	Belgium	50.55	4.74	2004–2006
CH-Oe2	Switzerland	47.29	7.73	2005
CN-Du1	China	42.05	116.67	2006
CW	China	35.25	107.68	2008–2009
DE-Geb	Germany	51.10	10.91	2004–2006
DE-Kli	Germany	50.89	13.52	2004–2006
DK-Fou	Denmark	56.48	9.59	2005
DK-Ris	Denmark	55.53	12.10	2004–2005
ES-ES2	Spain	39.28	-0.32	2004–2006
FR-Aur	France	43.55	1.11	2005
FR-Gri	France	48.84	1.95	2005–2006
FR-Lam	France	43.50	1.24	2005
GT	china	36.52	115.13	2009
HFK	Korea	34.55	126.57	2004–2006
IE-Ca1	Ireland	52.86	-6.92	2004–2006
IT-BCi	Italy	40.52	14.96	2004–2006
IT-Cas	Italy	45.07	8.72	2006
KR-Hnm	Korea	34.55	126.57	2004–2006
MSE	Japan	36.05	140.03	2001–2003
MY	China	40.63	117.32	2008–2009
NL-Lan	Netherlands	51.95	4.90	2005–2006
NL-Lut	Netherlands	53.40	6.36	2006
SX	China	32.56	116.78	2005
TC	China	35.08	109.07	2008–2009
TW-Tar	China	24.03	120.69	2006–2007
UK-ESa	United Kingdom	55.91	-2.86	2003–2005
UK-Her	United Kingdom	51.78	-0.48	2006
US-ARM	United States	36.61	-97.49	2003–2006
US-Bo1	United States	40.01	-88.29	2000–2007
US-Bo2	United States	40.01	-88.29	2004–2006
US-IB1	United States	41.86	-88.22	2005–2007
US-Ne1	United States	41.17	-96.48	2001–2005
US-Ne2	United States	41.16	-96.47	2001–2005
US-Ne3	United States	41.18	-96.44	2001–2005

^a^ Lat is latitude and lon is longtitude.

Although it is ideal to measure LE using the EC technique, the sum of H and LE was overall less than the available energy. EC sites underestimated LE, and an H flux was generally observed over most EC sites in Europe and North America [25]. Twine et al. (2000) [26] also reported the energy closure problem based on the Southern Great Plains 1997 Hydrology Experiment; the closure issue became more significant upon consideration of the long-term water balance. Therefore, we used the method provided by Twine et al. to eliminate the effect of the unclosed energy problem.

$$LE_{c}=\frac{\left(R_{n}-G\right)}{\left(LE_{o}+H_{o}\right)}\times LE_{o}$$ (1)

where LE_c_ is the corrected LE flux; H_o_ and LE_o_ are the uncorrected sensible heat flux and LE flux, respectively.

NDVI and FPAR values for the EC sites were derived from AVHRR products. The AVHRR NDVI data were downloaded using the ftp link (http://ecocast.arc.nasa.gov/data/pub/gimms/). LAI values were obtained from the GLASS product. GIMMS NDVI data at a 0.833-degree spatial resolution were used for model verification at the EC sites. FPAR data were calculated using the method proposed by Myneni and Williams (1994) [27]; this method was verified in various ecosystems [28]. The GLASS data can be found on the web site.

### 2.2. Satellite and reanalysis datasets

When estimating LE at regional scale for the physically based LE algorithms, we obtained AVHRR products, including bimonthly GIMMS NDVI and UMD Land Cover data with 1 km spatial resolution. We used the MODIS products with 0.05-degree spatial resolution, including annual land-cover type (MCD12C1). We also used the monthly LAI product (GLASS01B01), with 0.05-degree spatial resolution, from the GLASS datasets. The digital elevation model (DEM) product was obtained from the SRTM30 dataset. The DEM data can be found on the web site (https://dds.cr.usgs.gov/srtm/version2_1/SRTM30/). We used MERRA datasets for 1982–2010 to obtain daily radiation and meteorological data, including Rs, Rn, RH, Ta, ΔT and Ws. The MERRA dataset is a NASA reanalysis for the satellite era that uses a new version of the Goddard Earth Observing System Data Assimilation System Version 5 (GEOS-5). It has been reported that the MERRA dataset produced comparable results for global and energy cycle research [29]. We obtained MERRA data from the Goddard Earth Science Data and Information Services Center web site (https://gmao.gsfc.nasa.gov/reanalysis/MERRA/).

Considering the different temporal and spatial resolutions of the input data, including daily MERRA (½ degree latitude × ⅔ degree longitude spatial resolution), bimonthly GIMMS NDVI (0.833-degree spatial resolution), UMD Land Cover data (1-km spatial resolution), and monthly MODIS (0.05 degrees), we averaged the daily MERRA data and bimonthly GIMMS NDVI to yield monthly data sets. For the land cover classification, we used the UMD Land Cover data before 2001 and MCD12C1 after that. We spatially interpolated coarse resolution MERRA data and GIMMS NDVI to the resolution of 0.05 degrees using a spatial linear interpolation scheme. Adjustments were made to ensure all inputs parameters in LE models had consistent temporal and spatial resolutions.

## Methods

### 3.1. LE algorithms

#### 3.1.1. MODIS algorithm (MOD16)

The MOD16 algorithm developed by Mu et al. (2007) [11] was based on the Cleugh et al. (2007) [30] version using the Penman–Monteith model. Several improvements were made by Mu et al. (2011) [12] as follows: (1) improving the calculation of the vegetation cover fraction with FPAR; (2) modifying the estimation of aerodynamic, boundary-layer, and canopy resistance; (3) calculating the soil heat flux using available energy; (4) calculating LE based on daytime and nighttime LE; (5) calculating evaporation from the wet canopy surface and transpiration from the dry surface; (6) calculating evaporation from the saturated soil surface and evaporation from the moist soil surface. Previous validation results showed that the MOD16 algorithm successfully improved LE estimation using 46 AmeriFlux sites [31]. The MOD16 algorithm was applied to produce MODIS terrestrial LE using the MODIS products and GMAO daily meteorological dataset.

#### 3.1.2. Revised remote sensing-based Penman–Monteith LE algorithm (RRS)

Previous studies showed that high air temperature significantly decreases leaf stomata, causing structural defects [32]. Therefore, Yuan et al. (2010) [33] revised the equations dealing with the temperature constraint by accounting for stomata conductance and energy allocation between the vegetation canopy and soil surface [34]. The Beer–Lambert law was also used to exponentially partition the net radiation between the canopy and soil surface. Model parameters across different vegetation types were set to be consistent to reduce the effects of misclassification. Validation results based on 23 EC flux tower sites in China provided by Chen et al. (2014) [35] showed that the RRS algorithm improved LE estimation compared to the MOD16 algorithm.

#### 3.1.3. Priestley–Taylor algorithm (PT-JPL)

Priestley et al. (2009) [13] modified the PM equation by reducing the complexity of the aerodynamic and surface resistance and adding empirical parameters to develop a simple LE algorithm. On this basis, Fisher et al. (2008) [14] created a bio-meteorological method to calculate actual ET (ET, equivalent to LE). The inputs of this model included net radiation (Rn), normalized difference vegetation index (NDVI), soil adjusted vegetation index (SAVI), maximum air temperature (Tmax), and water vapor pressure (ea). The total ET includes the canopy transpiration and evaporation from soil and rain water intercepted by the canopy. Each component flux is determined by eco-physiological factors or conditions to reduce potential LE to actual LE using the Priestley–Taylor equation.

#### 3.1.4. Modified satellite-based Priestley–Taylor algorithm (MS-PT)

To overcome the computational complexities of aerodynamic resistance parameters, Yao et al. (2013) [36] revised the Priestley and Taylor equation using an index of soil water deficit calculated using the apparent thermal inertia and diurnal temperature range to constrain soil evaporation and using the revised linear two-source model (N95) to calculate vegetation transpiration. For canopy interception, the relative surface wetness index was modified to the fourth power of the soil moisture constraint. The model only required four inputs, including Rn, Ta, DT, and NDVI. Validation by Yao et al. (2015) [37] showed that the MS-PT algorithm had good performance over global forest biomes. Feng et al. (2015) [38] found that the MS-PT algorithm improved LE estimation over global grassland, shrubland and savanna.

### 3.2. Statistical analysis

We compared these models at the global scale using the modern-era retrospective analysis for research and applications (MERRA) dataset, the Normalized Difference Vegetation Index (NDVI) and Fractional Photosynthetically Active Radiation (FPAR) from the moderate resolution imaging spectroradiometer (MODIS) products, the leaf area index (LAI) from the global land surface satellite (GLASS) products, and the digital elevation model (DEM) from the shuttle radar topography mission (SRTM30) dataset to generate LE at the regional scale during 1982~2010.

The four models were first validated at 34 EC towers. We calculated the daily LE using the meteorological variables collected from EC sites and the NDVI and LAI from the satellite data (GIMMS NDVI and GLASS01A01). The vegetation index at the EC sites was calculated by computing the average of the surrounding pixels. Before the comparison, we calculated the coefficient of determination (R^2^), RMSE (root mean square error), and average bias for estimated LE to evaluate the model performances. We used Taylor diagrams [39] to analyze the performance of the four LE models. The Taylor diagrams provided a summary of how well patterns matched observations in terms of statistical indexes. These statistical indexes included standard deviations, centered root mean square error and their correlation. The radial distance from the origin indicated the standard deviations; the cosine of the azimuth angle showed correlation. The standard deviation of simulated patterns is shown to be proportional to the radial distance from the observed point to the patterns.

For regional estimates of LE, we applied simple spatially interpolated method to the MERRA data and AVHRR data to match the MODIS spatial resolution. Daily MERRA data and half monthly AVHRR NDVI data were combined to yield monthly data. After the interpolation process, we calculated LE over a cropland biome from 1982 to 2010 based on four models. The bias computed by the LE values based on one model minus that of the others was also calculated.

To estimate the long trend variability of LE, we calculated annual LE using four models and applied a linear trend analysis [40] using a linear model (y = ax + b), where t, a and b are the time, slope and intercept of the regression line, respectively. Student's t-test was used to analyze and classify the significance of the trend at the 1% and 5% level.

## Results

### 4.1. Models evaluation

**Fig 1:**
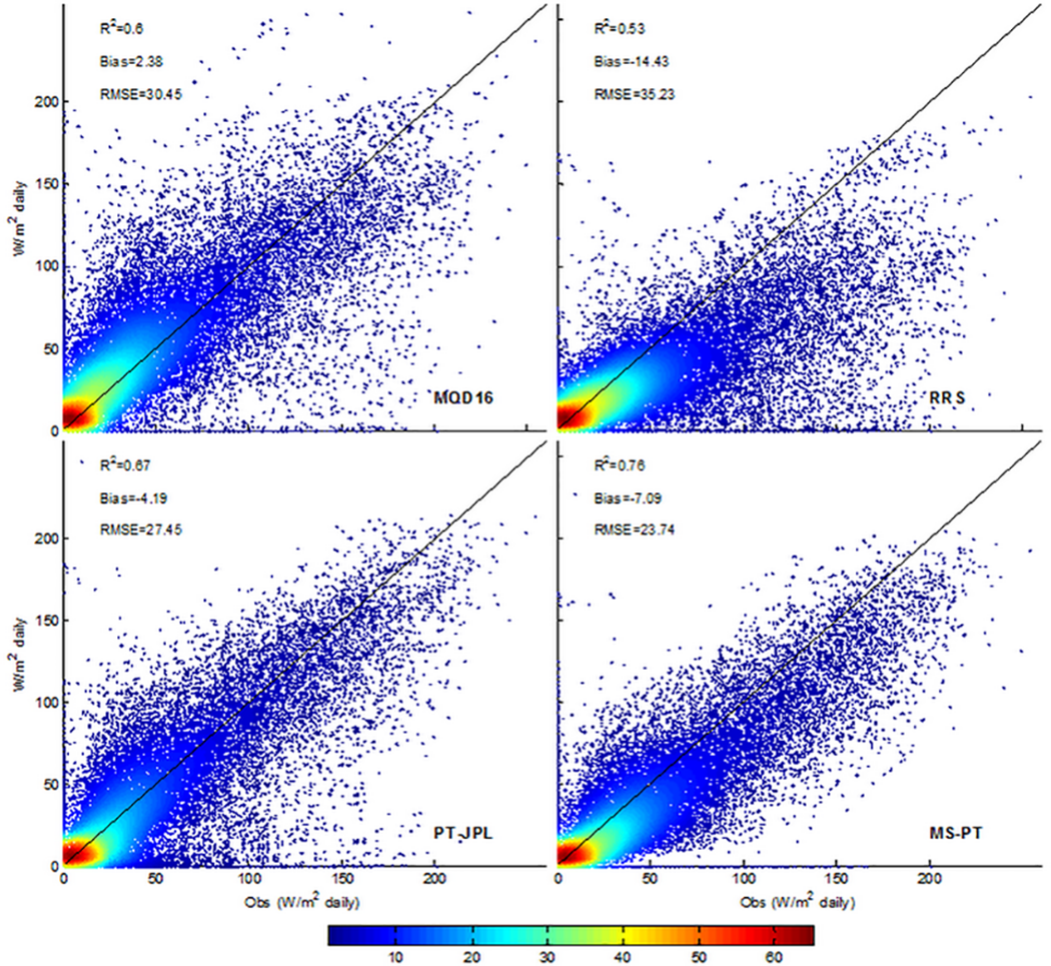
LE observed at the EC sites and predicted by the MOD16, RRS, PT-JPL and MS-PT algorithms during the period of 2000–2009.

All four models explained 50%~76% of the LE variability over all sites (Fig 1). The MS-PT showed the highest R^2^ (0.76). The averaged bias for all four models ranged from -14.9 to 2.38 W/m^2^. Generally, MOD16 showed a slight positive bias and the remaining methods had negative biases. RRS had the highest negative bias (14.43 W/m^2^) compared with the other models. The RMSEs for the four models ranged from 23 to 35 W/m^2^. The MS-PT showed the lowest RMSE (23.74 W/m^2^) compared with the other methods.

**Fig 2:**
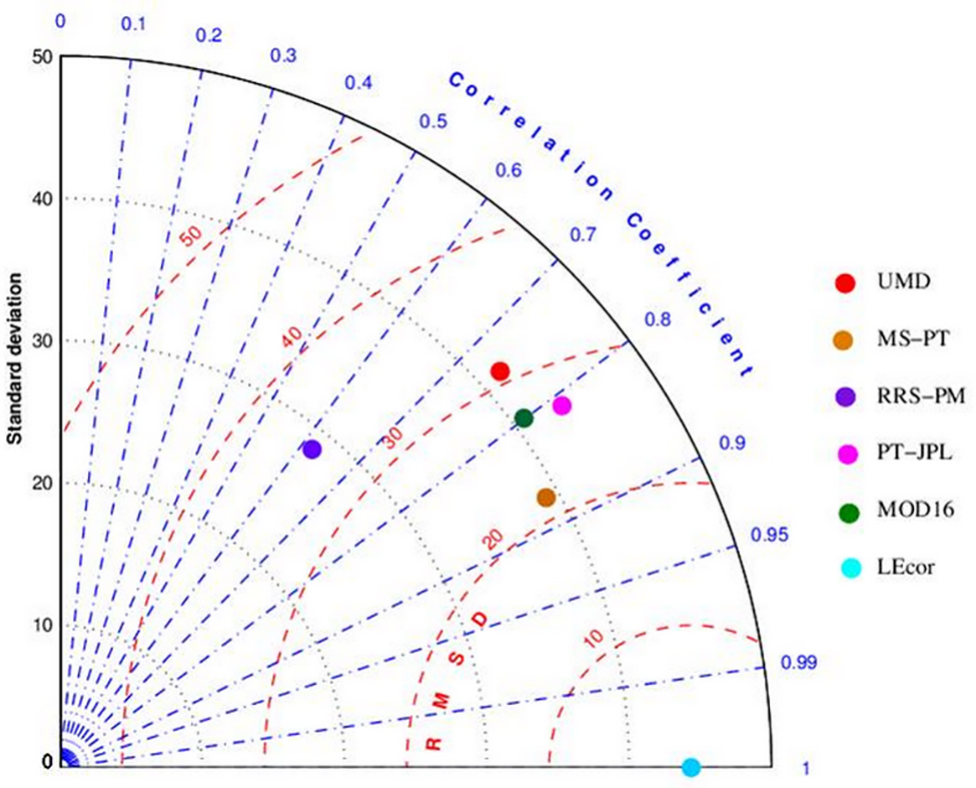
Taylor diagram for LE estimates using four LE algorithms driven by EC sites data. Six points were plotted on the semi-polar-style graph, with the circle representing the four models and field observation.

Generally, MS-PT had good overall performance for cropland (Fig 2). This result was consistent with the previous study[36]. In Fig 2, field observation had a standard deviation of 45.95 W/m^2^. The MS-PT lied close to the origin (field observation) and had a standard deviation of 40.04 W/m^2^. The correlation coefficient between the observation and MS-PT was 0.87, and the centered pattern RMS difference between the MS-PT and field observation was 22.44 W/m^2^.

### 4.2 Spatial patterns of LE

**Fig 3:**
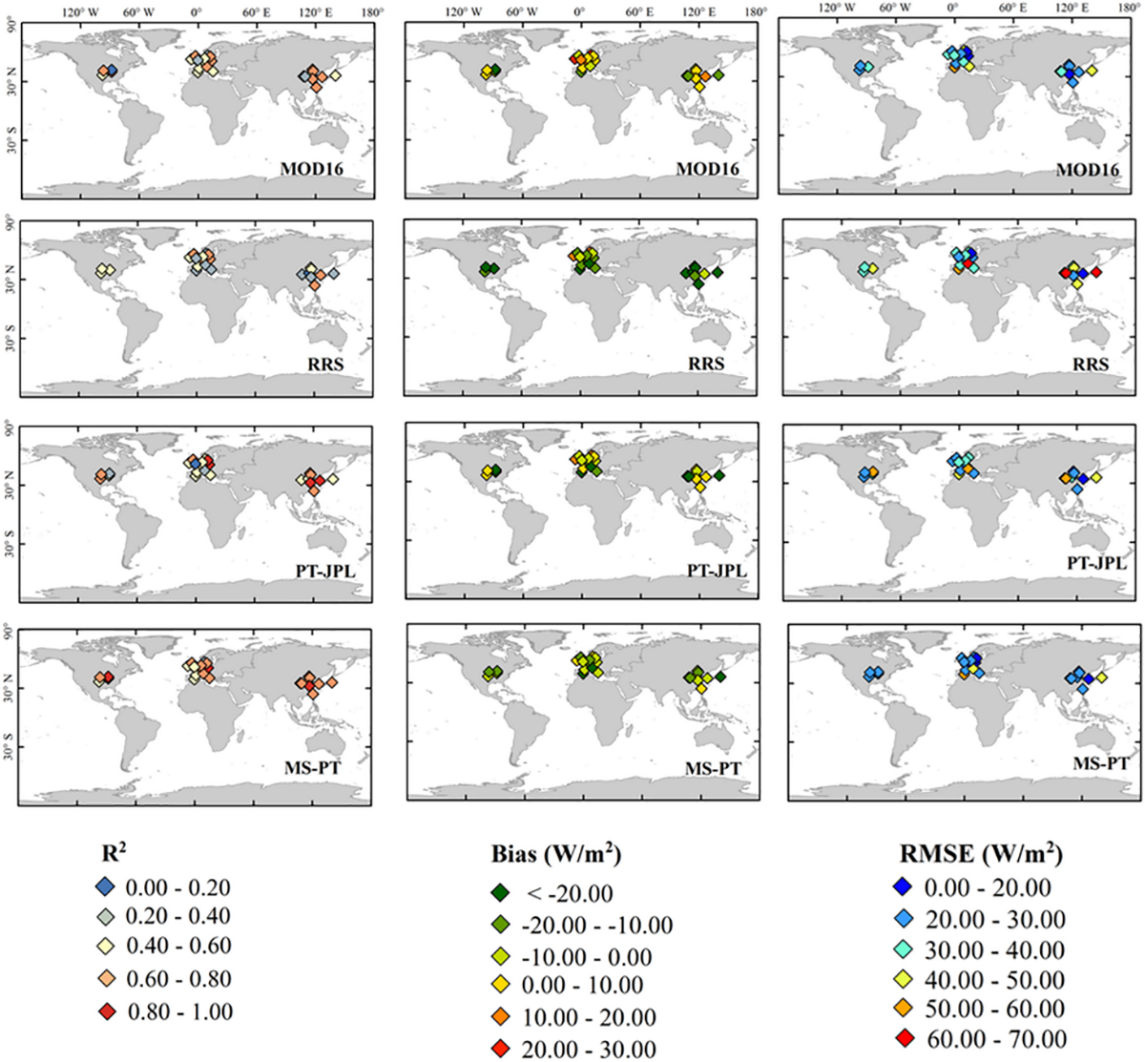
Spatial distribution of the correlation coefficients, RMSE and Bias for estimated daily LE calculated by MOD16, RRS, PT-JPL and MS-PT over the period of 2000–2009 (S1 File).

All models generally correlated well with ground observations of daily LE at most sites [17], with R^2^ ranging from 0.4 to 0.9, biases ranging from 15 to 50 W/m^2^, and RMSE ranging from 20 to 50 W/m^2^ (Fig 3). LE estimates by the four models showed high correlations with ground observations in humid mild regions such as east of Asia and central Europe, whereas low in arid and cold regions such the Mediterranean region and west of Asia. Compared with other methods, PT-JPL and MS-PT were more identical with ground observations [36, 38], especially at East Asia sites. RRS, PT-JPL and MS-PT had negative bias ranging from 10 to 20 W/m^2^ at most sites. MOD16 produced slight positive bias (about 10W/m^2^) at most sites. MS-PT had low RMSE ranging from 10 to 40 W/m^2^ compared with other models.

**Fig 4:**
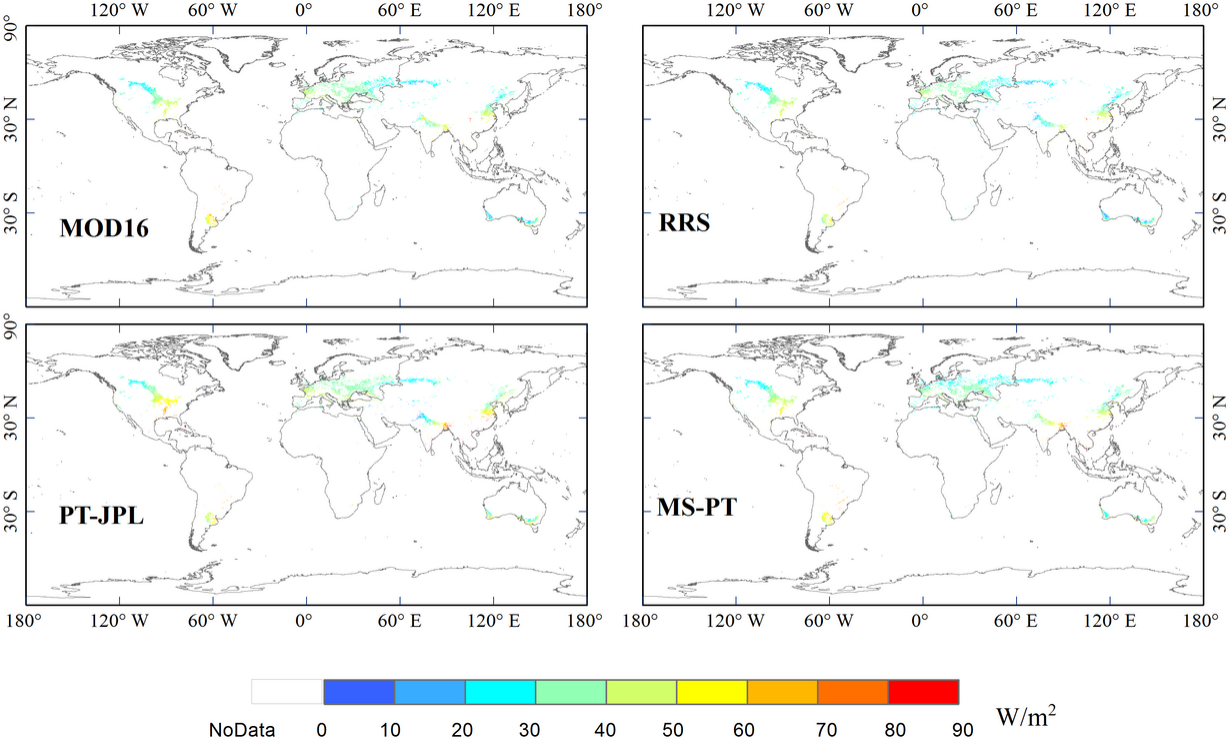
Estimated annual global terrestrial LE for cropland, averaged for 1982~2010 at a spatial resolution of 0.05°, from the four models, driven by MERRA data.

**Fig 5:**
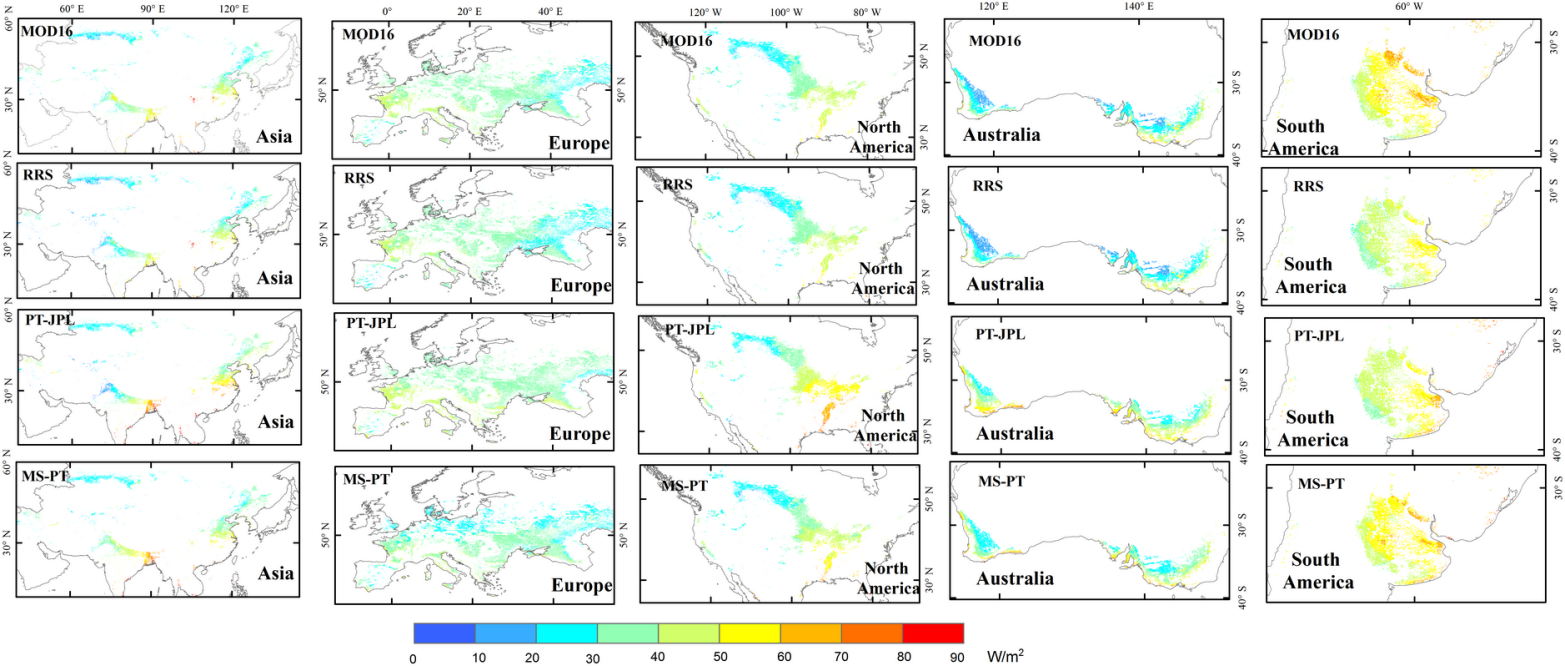
Estimated annual LE for cropland, averaged for 1982~2010 at different regions, with a spatial resolution of 0.05°, from the four models, driven by MERRA data.

Generally, the four models showed similar spatial patterns of LE, with high LE in warm and humid areas and low LE in arid and cold areas (Fig 4). However, some substantial differences in LE estimates existed among the models. The average annual LE from MS-PT was 36.26 W/m^2^, higher than RRS (33.49 W/m^2^) and MOD16 (35.63 W/m2) and lower than PT-JPL (39.73 W/m^2^). East of the Ural Mountains near Chelyabinsk, all models showed low LE estimates ranging from 20~50 W/m^2^ (Fig 5). In the northwest part of India, the LE was relatively low (30~40 W/m^2^), but in the southeast part, the LE was approximately 50~80 W/m^2^. This might be attributed to the spatial heterogeneity of available energy and humidity. The largest difference in LE estimates occurred between the eastern and western parts of Europe. The LE values in the western part of Europe (40~60 W/m^2^) were relatively higher than those in the eastern part (20~40 W/m^2^). This was likely because the water availability with large-scale atmospheric circulation influenced LE distribution. All models showed a low LE estimate (20~40 W/m^2^) in the northern part of North America and high LE estimate (40~70 W/m^2^) in the southern part.

**Fig 6:**
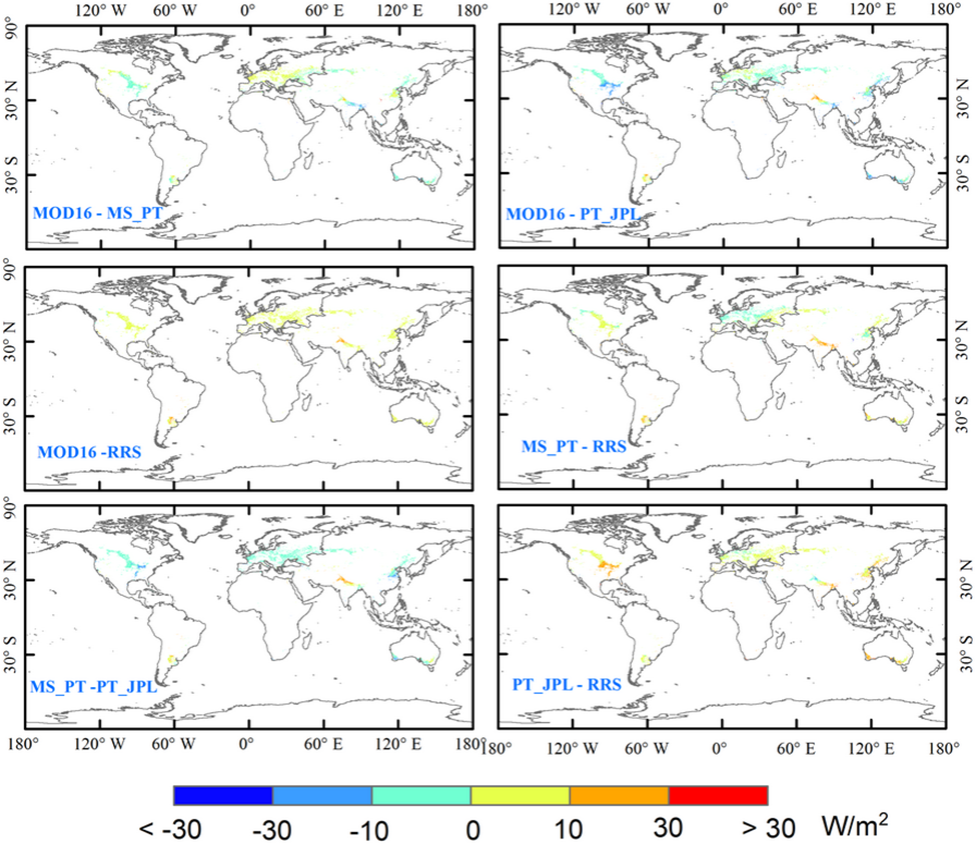
The differences between the four models, averaged during 1982~2010 at a spatial resolution of 0.05° from the algorithms, driven by MERRA meteorology data.

We further analyzed the spatial variability of magnitude of inter-model differences in the LE estimates in different parts of the world (Fig 6). MOD16 showed higher LE than MS-PT in the northwest part of North America (approximately 0~10 W/m^2^). However, MOD16 generated lower LE than MS-PT in the southeast part of North America (approximately 0~10 W/m^2^). MOD16 showed higher LE values than MS-PT (approximately 0~10 W/m^2^) in Western Europe. On the contrary, LE estimates by MOD16 were lower than those by MS-PT in Eastern Europe. LE estimates by MOD16 were lower than those by MS-PT in Asia, except in Eastern China. Small discrepancies in LE (-10~10 W/m^2^) were produced by MOD16 and MS-PT in South America. MOD16 produced higher LE estimates than RRS (10~30 W/m^2^) in all regions. Compared with MOD16, PT-JPL showed higher LE estimates (10~30 W/m^2^), especially in central North America. However, the LE estimates by PT-JPL were lower than those by MOD16 (10~30 W/m^2^) in North India. MS-PT predicted lower LE than PT-JPL (approximately 10~30 W/m^2^) in all regions, except the northern part of India. Compared with RRS, MS-PT showed lower LE estimates than RRS in Europe and the eastern part of China. On the contrary, in Northern India, the LE estimates by MS-PT were higher than those by RRS, with the difference ranging from 30 to 70 W/m^2^. These results indicated that the differences of four LE models are not spatial homogeneous. This might be attributed to different sensitivity of input parameters of LE models in different climatic regimes [41].

### 4.3 Annual mean and long-term trends

**Fig 7:**
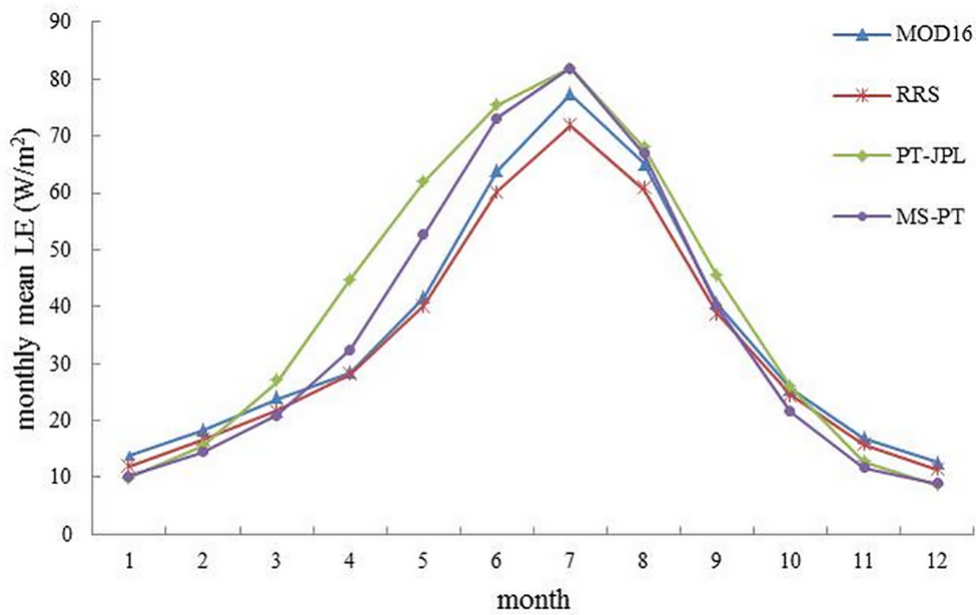
Comparison between the seasonal patterns of LE (W/m^2^) obtained by MOD16, RRS, PT-JPL and MS-PT.

**Fig 8:**
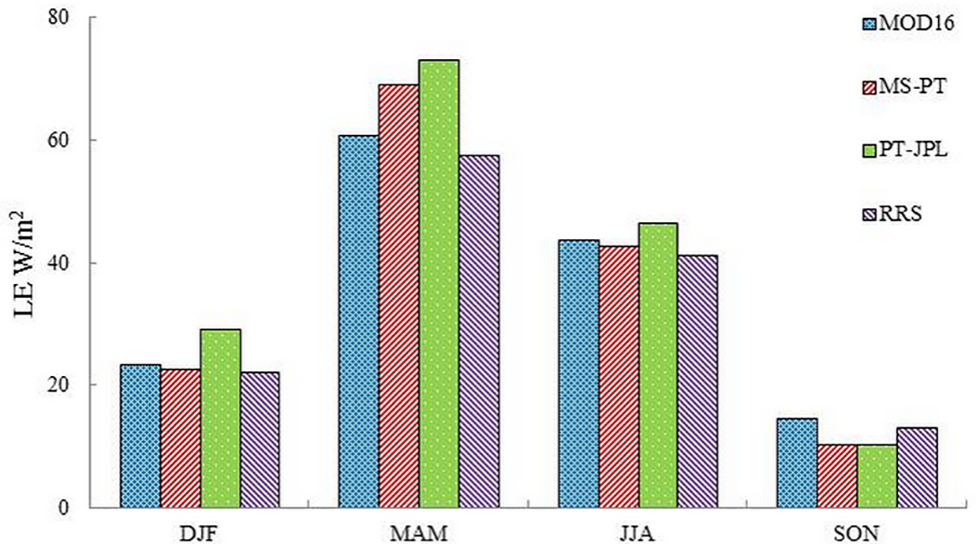
Seasonal cropland average LE during 1982~2010. DJF represents December, January, and February. MAM represents March, April, and May. JJA represents June, July, and August. SON represents September, October, and November.

All models showed strong seasonality in LE, and they captured the magnitudes and seasonal variations of LE (Fig 7). The results indicated that the LE produced by the four models had similar monthly patterns, with maxima in the summer (62~85 W/m^2^) and minima in the winter (11~42 W/m^2^). However, there were also substantial differences among models. The PT-JPL predicted the highest LE compared with the other models in spring and summer. The difference between MS-PT and PT-JPL mainly occurred in spring, when the temperature changed significantly (Fig 8). MOD16 and RRS almost showed similar magnitudes, with a slight difference occurring during summer months.

**Fig 9:**
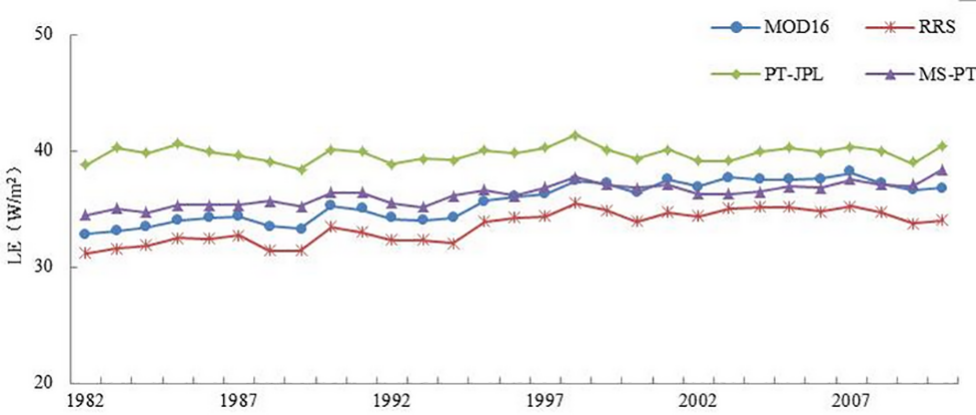
Interannual variations of average LE over the cropland ecosystem during 1982–2010 predicted by the four models.

Similar slightly annual increasing trends in LE estimates from 1982 to 2010 were produced by the four models (Fig 9). The LE estimates predicated by MOD16, RRS, MS-PT and PT-JPL were in the range of 31~42 W/m^2^.

**Fig 10:**
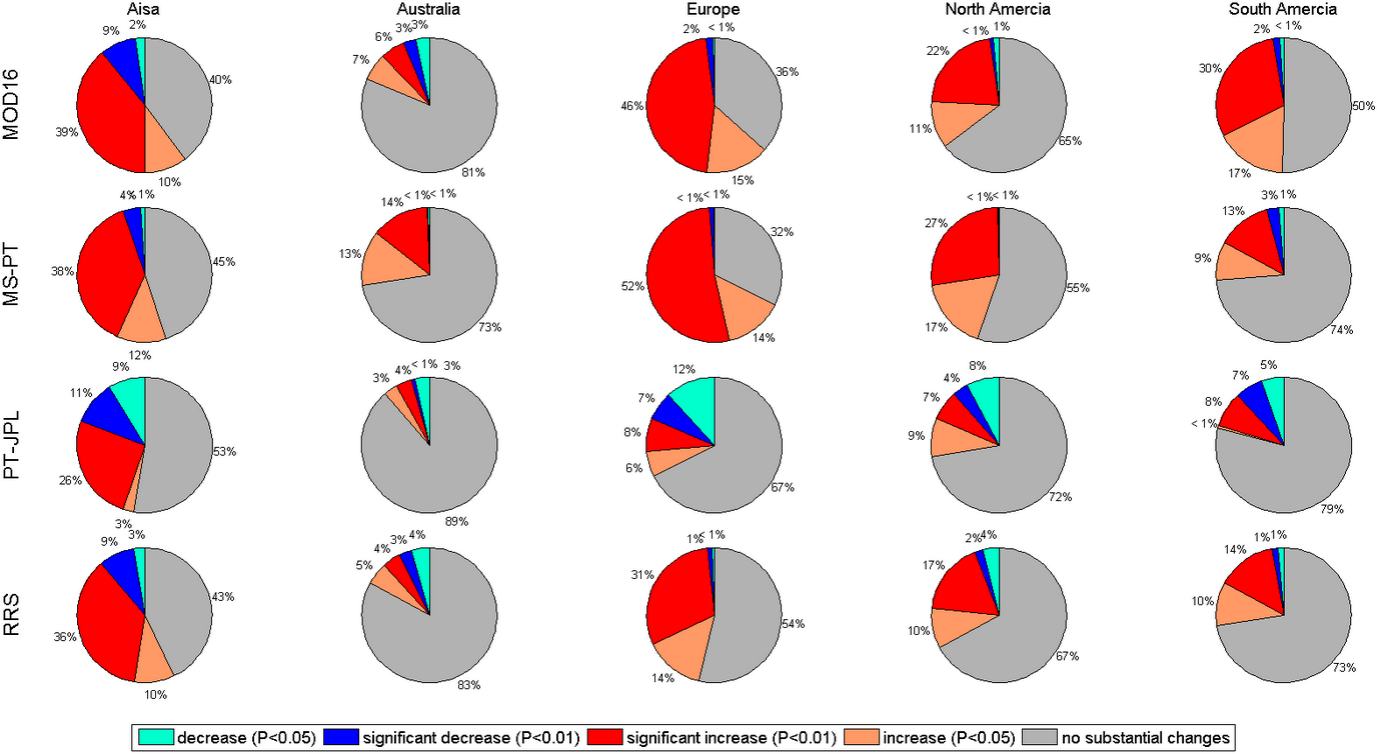
Different weight of long-term cropland LE trends of four models from 1982 to 2010.

**Fig 11:**
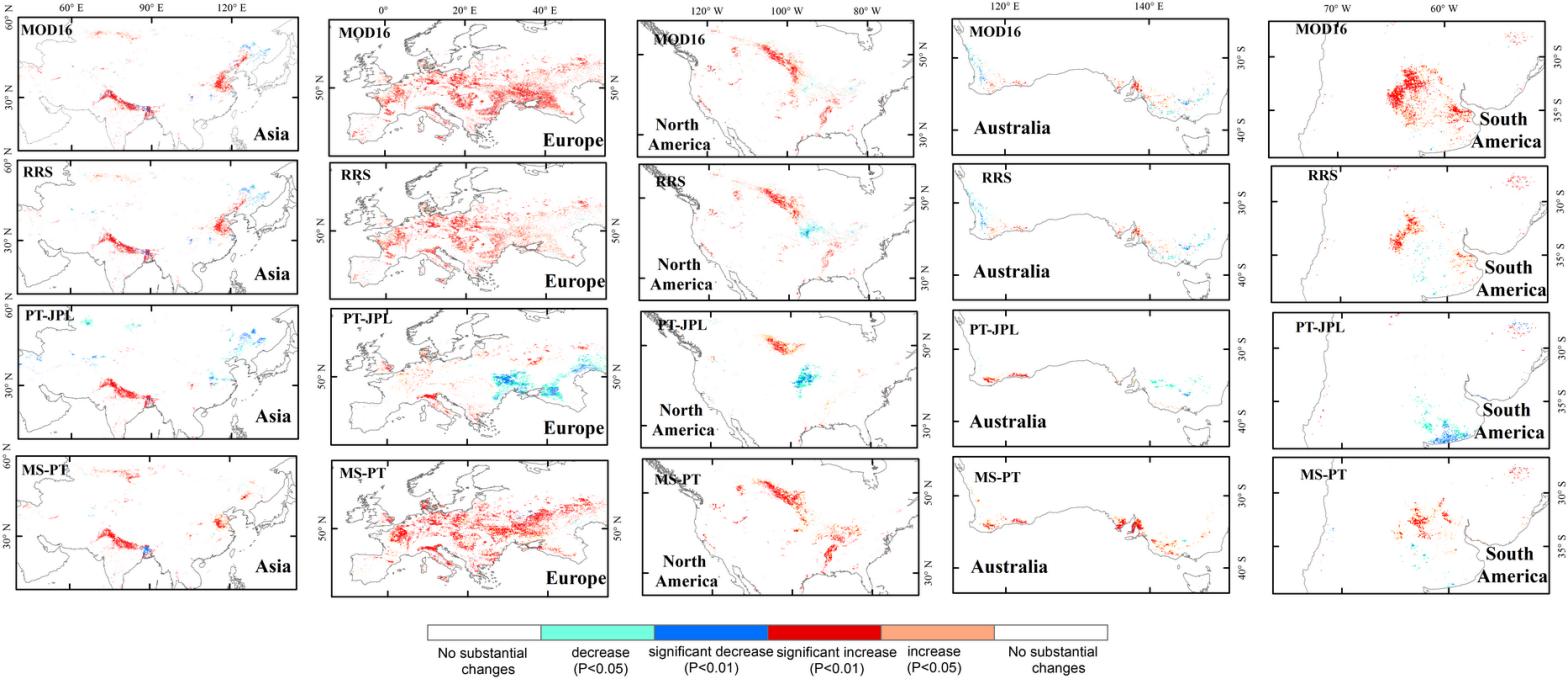
Spatial patterns of long-term cropland LE trends of four models from 1982 to 2010.

The long-term changes in LE differed among the four models and had different spatial patterns among the four models (Fig 10). All models showed strongly increasing LE in Asia. Specifically, approximately 46, 49 and 50% of area in Asia were dominated by the increasing trend in RRS, MOD16 and MS-PT, respectively. However, only 29% of the area was dominated by the increasing trend in PT-JPL. Generally, all models showed an increasing trend in Northern India (Fig 11). However, in the northeast part of China and east of the Ural Mountains, MOD16, RRS and MS-PT showed an increasing trend, while the opposite was found for PT-JPL, with a slightly decreasing trend. In the Far East Areas of Russia, MOD16, RRS, and PT-JPL simulated strongly positive LE trends; however, MS-PT showed negative LE trends. In Europe, increasing trends in LE accounted for approximately 61, 66 and 74%, according to MOD16 and MS-PT. However, only 45% and 14% of the area was dominated by the increasing trends in RRS and PT-JPL. Consistent LE increasing trends were found among the four models for Western Europe. PT-JPL simulated a slightly decreasing trend in Eastern Europe, while increasing ET trends were indicated by the other four models. In North America, increasing trends in LE accounted for approximately 16, 27, 33 and 44%, according to the PT-JPL, RRS, MOD16 and MS-PT, respectively. In the central south of Canada, all models showed an increasing trend. In Iowa, RRS and PT-JPL showed a decreasing trend. In Australia, no substantial changes were found by the four models. In South America, only MOD16 showed increasing trends in LE, which covers approximately 47% of the total area in South America. There is an obvious difference of annual trend between PT-JPL and the other three models. These differences have connection with climate regime and local climate condition via meteorological parameters such as relative humidity and diurnal temperature range. These meteorological parameters have closely relationship with constraint parameters in these algorithms. The MOD16 and RRS generally adopt these meteorological parameters to calculate canopy resistance and soil resistance but PT-JPL and MS-PT adopt these parameters to calculate scale factors to downscale potential LE to actual LE. Relative humidity is used as input in PT-JPL but no used in MS-PT. So the variations of meteorological parameters related to climate regime cause the difference of annual trend cause the difference of annual trend among the models.

## Discussion

### 5.1. Model performance

Ground measurements and estimated LE were in good agreement over cropland sites, suggesting that it can be considered as a suitable method for evaluating the cropland LE over a variety of climate conditions. The reason might be reduced the number of input parameters, which only included net radiation, NDVI, air temperature and diurnal temperature range. The fewer input parameters eliminated the effect caused by input uncertainties. The MS-PT also avoided the complex calculations of aerodynamic and surface resistance. The good performance of MS-PT was similar to that of our previous studies [36–38]. Yao et al. (2014) [42] also found that the MS-PT model improved the LE estimation compared with the 40 EC sites data.

Both MS-PT and PT-JPL were developed based on a simple LE equation for radiation- and temperature-based equilibrium by Priestley and Taylor [13]. However, the different performances of the two models might be caused by the different calculations of the ecophysiological constraint functions. Fisher et al. (2008) [14] calculated relative surface wetness by squaring relative humidity (RH) twice. However, for MS-PT, the relative surface wetness was the fourth power of the soil moisture constraint. The soil moisture constraint in MS-PT was the exponential algorithm of the Apparent Thermal Inertia (ATI). The ATI was simply estimated as the inverted diurnal temperature range. For PT-JPL, the soil moisture constraint was the exponential algorithm of RH and saturation VPD. Therefore, appreciable differences might occur due to the calculation of actual soil surface evaporation and actual vegetation transpiration.

The results of MOD16 were generally concordant with the results of RRS. However, there were small discrepancies in the LE estimates by the two models. This difference might be attributed to the difference in model structures related to the calculations of surface resistance. Both LE models used the same controlling factors from VPD and minimum temperature (Tmin) on stomatal conductance. Mu et al. (2011) [12] calibrated VPD and Tmin for each biome. However, these variables were different in RRS. Both models used different ways to partition the net radiation between the canopy and soil surface. For MOD16, the fraction of absorbed photosynthetically active radiation was a proxy of the vegetation cover fraction. However, Fc was calculated using the Beer–Lambert law with LAI. All these factors led to different results of LE predicted by the two models.

### 5.2. Spatial and temproal differences

The satellite estimates of LE predicted by these LE models generally displayed a similar distribution, with large values at low latitudes but smaller values at high latitudes. This was probably due to uneven distribution of the solar radiation. In North America, LE values in the northern part were lower than those in the southern part. A similar pattern occurred in East Asia and North India. Vinukollu et al. (2011) [43] calculated LE using three models, including the Surface Energy Balance System (SEBS), MOD16 and a modified Priestley–Taylor approach (PT-JPL), and found a similar distribution pattern. Zhang et al. (2010) [44] quantified global terrestrial ET using the modified Penman-Monteith approach from 1983 to 2006 and found strong regional variations and latitudinal gradients corresponding to global climate patterns. The results also showed that coastal areas had higher LE than land areas, such as Europe and South Australia.

Inter-model differences generally ranged from -30 to 10 W/m^2^ based on MERRA data for 1982~2010. Although these LE models were developed based on a physical mechanistic equation, different results might be attributed to the model structure and sensitivity of input variables, which were related to the regional climatic environment. Previous studies also supported our conclusion and highlighted the model structure and the uncertainty of input data. Behrangi et al. (2014) [45] compared MOD16 and PT-JPL and found that PT-JPL values were larger than MOD16 values. Vinukollua et al. (2011) [43] reported higher EC tower LE values of PT-JPL than MOD16 by estimating terrestrial latent heat flux using a single-source energy budget model, MOD16 and PT-JPL.

In this study, global decadal cropland LE was calculated using four process-based LE models. We found an increasing trend of global average annual LE and different variations of LE in specific regions over the cropland ecosystem. Numerous studies showed increasing transpiration due to the increase of the growing seasons in recent decades [46–50]. The increasing trend of cropland LE might be attributed to increasing irrigation water diversions. McLaughlin et al. found an increasing trend of irrigation water from 1960 to 2010. The regional long-term trend of LE generally varied with changes of anthropogenic activities and regional climate. Sacks et al. (2011) [51] analyzed corn and soybean data over the US corn belt from 1981~2005 and found that the annual-average LE increase was 0.50 W/m^2^, or 6.3 mm/year, over the 25 years. Riediger et al. (2014) [52] used a framework of the comprehensive research cluster Nachhaltiges Land management in Norddeutschen Tiefland and predicted increasing scenarios in ET at Uelzen in Germany.

### 5.3. Uncertianties analysis

Uncertainties in predicting LE were considered to be largely induced by the forcing data [53–55]. Liu et al. (2015) [54] analyzed the impact of input data uncertainties on (ET) using the Climate Research Unit (CRU) TS3.1 data, the European Centre for Medium-Range Weather Forecasts (ECMWF) ERA-Interim Reanalysis, the National Centers for Environmental Prediction/National Center for Atmospheric Research (NCEP/NCAR) reanalysis data, the Global Meteorological Forcing Data set for land surface modeling from Princeton University, and MERRA data for 1979–2008 in Northern China. They found that MERRA displays the lowest correlation with measurements for temperature, wind and relative humidity. These uncertainties might affect the accuracy of the final calculation. Zhao et al. (2013) [56] also reported that MERRA surface solar radiation has an average bias error of +20.2 W/m^2^ at different time scales based on American FLUXNET sites. NDVI or LAI were often used as a surrogate of vegetation moisture and for the calculation of vegetation coverage. However, the uncertainties in NDVI or LAI led to erroneous subsequent calculation of canopy conductance.

In addition to the uncertainties in forcing data, another source of uncertainty might stem from the scale mismatch between the remote sensing data and the EC observations. McCabe et al. (2006) [57] used three different remote sensing data from Landsat (60 m), ASTER (90 m), and MODIS (1020 m) to study the effects of sensor resolution on land surface flux estimates. These scholars found that MODIS-based flux retrievals could not effectively characterize the different latent heat flux patterns between soybean and corn. Kustas et al. (2004) [58] also found a lack of spatial variability in the coarse resolution data-based LE retrievals compared with different pixel resolutions of remote sensing inputs.

## Conclusions

In this study, four LE satellite-based LE algorithms, including MOD16, RRS, PT-JPL and MS-PT, were evaluated over a cropland ecosystem based on 34 EC flux towers sites. We compared these LE model performances with ground LE measurements and satellite forcing data. All models had different assumptions, data requirements, empirical model parameters and model structures ranging from complex Penman-Monteith and energy balance schemes to simpler Priestley–Taylor approaches. The results showed that all the LE models produced acceptable results for cropland ecosystems. MS-PT produced the best performance with respect to LE prediction, with a higher R^2^, a lower bias and a lower RMSE. MOD16 and RRS showed the lowest LE retrievals compared with the other models.

All algorithms had high LE at low latitudes and low LE at high latitudes. The results showed that the magnitudes and seasonal variations of LE were well characterized by the four models. A slightly increasing trend in the annual average LE from 1982 to 2010 was also observed. Considering that uncertainties still existed, the limitations associated with the model comparison, as analyzed above, might also cause modeling errors. Intercomparison of models over a range of diverse bioclimates and longer periods are recommended for future studies.

## Supporting information

S1 File The site statistics of four models. The estimated daily LE calculated by MOD16, RRS, PT-JPL and MS-PT over the period of 2000–2009 were contained in the site_stat2.rar. The model validation results were in each Excel tables (.XLSX)
(RAR)
